# Environmental and lifestyle factors and risk of thyroid carcinoma: a cross-sectional study

**DOI:** 10.1007/s12020-025-04441-2

**Published:** 2025-10-06

**Authors:** Valentina Sada, Alessia Cozzolino, Ilaria Neri, Ritamaria Di Lorenzo, Livia Barba, Roberta Modica, Pasquale Dolce, Oumaima Achour, Carlotta Pozza, Elisa Giannetta, Daniele Gianfrilli, Lucia Grumetto, Valeria Ascoli, Claudio Bellevicine, Giancarlo Troncone, Andrea M. Isidori, Annamaria Colao, Antongiulio Faggiano

**Affiliations:** 1 Department of Experimental Medicine, Sapienza University of Rome, Rome, Italy; 2 Xenoanalysis Laboratory, Pharmacy Department, University of Naples Federico II, Naples, Italy; 3 National Laboratory on Endocrine Disruptors, Consorzio Interuniversitario, National Institute of Biostructures and Biosystems (INBB), Rome, Italy; 4 Unit of Endocrinology, Azienda Ospedaliera di Rilievo Nazionale A. Cardarelli, Naples, Italy; 5 Unit of Endocrinology, Department of Clinical Medicine and Surgery, University of Naples Federico II, Naples, Italy; 6 Biostatistics and Clinical Trial Methodology Unit, Clinical Research Center DEMeTra, Department of Translational Medical Science, University of Naples Federico II, Naples, Italy; 7 Department of Translational Medical Science, University of Naples Federico II, Naples, Italy; 8 Department of Radiological Oncological and Pathological Sciences, Sapienza University of Rome, Rome, Italy; 9 Department of Public Health, University of Naples Federico II, Naples, Italy; 10 Centre for Rare Diseases (ENDO-ERN accredited), Policlinico Umberto I, Rome, Italy; 11 Department of Clinical and Molecular Medicine, Endocrine-Metabolic Unit, Sapienza University of Rome, Sant’Andrea University Hospital, Via di Grottarossa, 1035 - 00189 Roma, Italy

**Keywords:** Thyroid nodules, Thyroid cancer, Endocrine disrupting chemicals, Obesity, Lifestyle - environment

## Abstract

**Purpose:**

The incidence of differentiated thyroid carcinoma (DTC) is increasing, and environmental factors, including lifestyle and endocrine-disrupting chemicals (EDCs) exposure, have been advocated as having an etiologic role. To investigate the relationship between DTC, EDCs exposure, and lifestyle in a population from two Italian regions.

**Methods:**

A cross-sectional study evaluating chemical exposure, lifestyle, and DTC diagnosis in subjects with thyroid nodules, undergoing Fine Needle Aspiration Cytology from May 2019 to February 2021. 193 patients were split into groups based on cytological diagnosis: group A, benign or low-risk (TIR2-TIR3A); group B, high-risk (TIR3B-TIR4-TIR5). Age, sex, weight, height, body mass index (BMI), waist circumference (WC), thyroid hormone profile, lifestyle variables, and serum EDCs levels were compared.

**Results:**

The percentage of male patients was significantly higher in group B (*p* = 0.033), as well as the prevalence of obesity (*p* = 0.001) and BMI (*p* = 0.013). Visceral obesity was higher in group B, without reaching statistical significance (70.4% vs. 58.7%, *p* = 0.252), while WC was significantly higher in group B (*p* = 0.008). EDCs serum levels were not significantly higher in group B. A multivariable analysis found a significant association between high-risk cytology and WC (OR 1.04, 95% CI 1.01–1.08, *p* = 0.027). Considering patients with obesity, a linear correlation was observed between WC and bis(2-ethylhexyl) phthalate (DEHP) levels (r:0.40; *p* = 0.034).

**Conclusion:**

Obesity could have a role in DTC development. Furthermore, the WC and DEHP levels correlation in patients with obesity supports the hypothesis of an interaction between EDCs exposure and adipose tissue excess in increasing DTC risk.

## Introduction

The incidence of differentiated thyroid carcinoma (DTC) is continuously increasing [1], involving not only micro-carcinomas but tumours of all sizes. A plateau has not been achieved despite the long-term use of sensitive diagnostic procedures [2]. These findings suggest that DTC increased incidence cannot be attributable only to the widespread use of ultrasonography and hints at an etiologic role in environmental pollution in addition to lifestyle factors. This hypothesis was empowered by the observation that increased incidence mainly involves DTC with BRAF and RAS mutations [3] which seem to occur more frequently in chemically polluted areas [4]. Despite the improved knowledge of DTC molecular genetics [5, 6], the aetiology of genetic damage is still unclear. In addition, genetic alterations such as RAS mutations and RET rearrangements have been detected in benign nodules. These are viewed as markers of precancerous lesions [5].

Endocrine-disrupting chemicals (EDCs) are mostly synthetic compounds present ubiquitously in the environment due to legal/illegal discharge because of their employment in the industrial production of plastic materials, food packaging, and personal goods [7]. Some EDCs, according to their lipophilicity, are not easily biodegraded and, therefore, bioaccumulated in the environment. EDCs can be widely absorbed by humans, mainly through diet, interfering with the endocrine system’s function [8]. The thyroid is a target of EDCs, which may affect both the metabolism and transport of thyroid hormones and their transcriptional activity [9]. Indeed, environmental pollution exposure has been associated with impairment of thyroid-hormone profile, autoimmune, and thyroid nodules [5, 8, 10]. EDCs have also been proved to play a role in the development of cancer [11]. The increasing environmental pollution has already been advocated to play an etiologic role in the rising incidence of DTC, exerting its toxicity even at low doses [6, 12], but data about their relationship to DTC risk are still scarce [6, 13–16]. Exposure to certain EDCs, such as polybrominated diphenyl ethers, phthalates and heavy metals, is associated with an increased risk of thyroid cancer, according to a recent meta-analysis of 15 studies mainly on non-European cohorts (10 from China, 4 from the USA and 1 from Norway). There was high heterogeneity between the studies, the small number of them involving each type of EDCs and the retrospective nature of some studies may have weakened the quality of the results [17]. Recent publications have reinforced the need for integrated research on EDCs and thyroid disease. A review of Gorini et al. on phthalates and non-phthalate plasticizers emphasized the ubiquity of human exposure, their capacity to interfere with the hypothalamic-pituitary-thyroid axis and thyroid receptor function, pointed out the paucity of human data linking these chemicals to thyroid nodules and carcinoma. Furthermore, they highlighted the heterogeneity of the restricted use of different EDC in the various parts of the world [18]. Finally, a recent cross-sectional study of Yu et al. based on more than 6,000 adults from NHANES (2007–2018) reports a possible correlation between per- and polyfluoroalkyl substances (PFAS) exposure and altered peripheral sensitivity to thyroid hormone and deiodinase activity, suggesting disruption of thyroid homeostasis even at background exposure levels. No relationship with thyroid cancer was evaluated [19].

Lifestyle, including diet, physical activity, smoking habits, and alcohol consumption, has been demonstrated to have a role in cancer development and progression. It has also been advocated in the development of DTC, even though some variables seem to act in opposite ways than expected [20].

Surprisingly, smokers seem to have a lower risk of developing DTC, probably due to the lower Thyroid Stimulating Hormone (TSH) levels and lower body mass index (BMI). It has been hypothesized that smoking activates the sympathetic system increasing free thyroxine and free triiodothyronine levels, reducing TSH levels [21].

Alcohol consumption seems having an inverse association with thyroid cancer risk [22, 23] and a lower prevalence of thyroid enlargement. Indeed, solitary nodules have been found in people with high alcohol consumption, with reduction of TSH levels as a possible explanation [24]. In animal studies, chronic ethanol treatment reduces the responsiveness of the hypothalamic-pituitary-thyroid axis to central stimulation, acting on the thyrotropin-releasing hormone neurons of the paraventricular nucleus [25].

The association between physical activity and DTC is poorly understood. A systematic review and meta-analysis, reappraising the relationship between physical activity, diabetes, and DTC, showed a significantly increased risk in patients with diabetes. Conversely, it did not show any association with physical activity [26].

Obesity and overweight are multifactorial disorders associated with the development of chronic diseases, such as cardiovascular disorders, diabetes, and increased risk of neoplasia, including DTC [27, 28]. In the last three decades, obesity has shown a pandemic development, leading Italy to an increased rate of 27.5% [29]. Notably, adipose tissue induces a chronic inflammatory microenvironment responsible for DNA damage leading to DTC [30]. Leptin, a hormone produced by adipose tissue, seems to improve cellular proliferation and survival, inhibiting apoptosis by stimulating thyrocyte receptors [31, 32].

This observational study aims to evaluate the possible relationship between lifestyle variables and exposure to various EDCs in patients from two different Italian regions, integrating these variables into a single analysis, to provide novel, hypothesis-generating insights on contributors to the increasing prevalence of DTC in the European population.

## Materials and methods

This was an analytical cross-sectional study evaluating chemical exposure, lifestyle, and thyroid carcinoma diagnosis in patients with thyroid nodules, with ultrasonographic risk features, submitted to Fine Needle Aspiration Cytology (FNAC) according to American Thyroid Association guidelines [33]. The study adopts a convenience sampling approach. This non-probabilistic method allows for rapid data collection but limits the general population’s representativeness.

From May 2019 to February 2021, 201 patients, residing in two Italian regions, Lazio and Campania, were enrolled by the Department of Experimental Medicine of Sapienza University of Rome and the Department of Clinical Medicine and Surgery of Federico II University of Naples. Inclusion criteria were: (a) age ≥ 18 years, (b) diagnostic thyroid cytology (TIR2-TIR5 SIAPEC-IAP categories [34]), (c) clinical management, including surgery, entirely performed in the involved centres. These criteria were chosen to enable a comparison between benign and malignant thyroid nodules, which was the study’s primary aim.

Exclusion criteria were: (a) nondiagnostic cytology (TIR1-TIR1C SIAPEC-IAP categories [34] as these cases do not allow reliable classification), (b) clinical and/or cytological and/or histological features of medullary thyroid carcinoma, which represents a distinct entity with different pathogenesis and management.

Cytological samples have been evaluated at the Units of Cytopathology of Sapienza and Federico II University according to SIAPEC categories [34]. All patients with TIR3B-TIR4-TIR5 cytological diagnosis received an indication of surgery, while TIR3A cytological diagnosis underwent further FNAC within 6 months and then followed-up or addressed to surgery, according to guidelines. Patients with TIR2 cytological diagnosis have been followed-up or addressed to surgery according to physician recommendation.

Demographic and anthropometric data [age, sex, weight, height, BMI, waist circumference (WC), thyroid hormone profile] were collected from all patients. Lifestyle variables were collected through a self-reported questionnaire assessing smoker status (present/former smoker/never), alcohol consumption (yes/no), and physical activity (yes/no).

Blood samples for EDCs evaluation were obtained at the time of enrolment, during the day of the FNAC execution. 5 mL were collected in Vacu-test^®^ tubes from the antecubital vein, centrifuged at 3000 rpm for 20 min, transferred in Eppendorf, and stored at -20^°^C until the analysis. Sample preparation was carried out adding to 300 µL of serum 150 µL of perchloric acid 25% w/v aqueous solution to precipitate the proteins, two volumes of a mixture of ethylacetate/n-hexane 50/50 v/v, 100 mg NaCl, and finally 15 µL of a 10 mg/mL solution of biphenyl in ethylacetate as internal standard. The dried supernatant was used for the detection of the selected panel of screened EDCs: Bisphenol A (BPA) and 6 of its structural analogues, Bisphenol F (BPF), Bisphenol E (BPE), Bisphenol B (BPB), Bisphenol S (BPS), Bisphenol AF (BPAF), bisphenol A diglycidyl-ether (BADGE), 1,4-dichlorobenzene (DCB), 1,2,4,5-tetrachlorobenzene (TCB), Triclosan (TCS), 2-chlorophenol, (2-CP), 4-Nonylphenol (4-NP), bis(2-ethylhexyl) phthalate (DEHP) and its main metabolite, mono-ethylhexyl phthalate (MEHP). Pharmacy Unit, Federico II University of Naples performed the extraction, identification and quantitation of the investigated analytes in human sera using an already validated method [35]. Serum analyses were performed using high-performance liquid chromatography (HPLC) in a certified laboratory and in accordance with standard operating procedures. Quality assurance and quality control measures included the use of blanks and triplicates spiked samples to verify accuracy and reproducibility. Detection and quantification limits along with coefficients of variation were reported for each analyte in the published article [35]. HPLC (LC-20 VP - Shimadzu Corp., Kyoto, Japan) coupled with tandem ultraviolet/fluorescence detection (UV/FLD) (ultraviolet (UV)–visible detector (Shimadzu Model SPD10 AV) and model RF-20, Shimadzu Corp., Kyoto, Japan) were employed. Any possible background contamination was avoided by keeping the plastic labware in contact with a solution 50:50 n-hexane–tetrahydrofuran for 3 h before their use [36]. The analyses were performed in triplicate, and the results are the averages of three determinations.

EDCs exposure has been also evaluated through a self-reported questionnaire assessing the residence in industrial areas or waste dumping sites (legal/illegal), type (industrial foodstuffs/organic food), cooking modes, and frequency of foods consumed during a standard week (more or less three times a week), in consideration of EDCs presence in polluted areas and of their use for food packaging.

The molecular analysis has been performed on cytology specimens of nodules, from TIR3A to TIR5 cytological categories at the Cytopathology Unit, Federico II University of Naples.

A dedicated FNAC pass has been collected and stored.

in Eppendorf with DNAsi-free solution at -20 °C, while waiting for the cytological report, to obtain nucleic acid with optimal quality and quantity.

An aliquot of the aspirated material has been suspended in a vial of nuclease-free water (Ambion, Invitrogen, Thermofisher, Waltham, MA, USA) and has been stored at -20 °C until the final cytological diagnosis was available. The DNA and RNA have been simultaneously extracted using the AllPrep DNA/RNA kit (Qiagen). The nucleic acid extracted from the vial has been analysed using a real-time PCR (RT-PCR)-based procedure on a Quant Studio 5 platform (Applied BioSystem, Thermofisher) using the Entrogen Thyroid Cancer Mutation Analysis Panel kit (EntroGen Inc, Woodland Hills, CA, USA) that detects *BRAF*V600E, *KRAS* codons 12 and 13, *NRAS* codon 61, *HRAS* codon 12, 13 and 61, and the *RET/PTC*1, *RET/PTC*3 and *PAX8/PPARg* fusions. The assay has been performed in two runs: one run to detect point mutations in the *BRAF* and *RAS* genes on DNA, and the other for the fusion genes on RNA. Fusion detection reactions have been performed with a one-step procedure that combines cDNA synthesis and RT-PCR. The resulting RT-PCR amplification curves have been visualized on QuantStudioDesign&Analysis software v 1.2 (Thermofisher).

### Study population

The study population has been divided into two groups: group A, patients with benign/low-risk cytological diagnosis (TIR2-TIR3A); and group B, patients with high-risk/malignant cytological diagnosis (TIR3B-TIR4-TIR5). This group stratification was performed by comparing the two groups according to the clinical management recommended by ATA guidelines [33]. Therefore, the TIR3A category was grouped with TIR2 category due to their alleged benignity for which clinical management is recommended, while the TIR3B category was grouped with TIR4 and TIR5 categories for which surgical management is recommended [33]. Moreover, patients who underwent surgery were further split into two groups: group 0, patients with a benign histological diagnosis, namely follicular adenoma, or nodular goiter (except for one case of Non-invasive follicular thyroid neoplasm with papillary-like nuclear features-NIFTP); group 1, thyroid carcinoma diagnosis.

Furthermore, to investigate the possible role of lifestyle and body weight gain in the development of thyroid cancer, the study population was clustered according to BMI, considering 30 kg/m^2^ as cut-off patients without obesity, BMI < 30 kg/m^2^, and patients with obesity, BMI ≥30 kg/m^2^.

### Statistical analysis

The statistical analysis was primarily descriptive and exploratory, as the study was based on a convenience sample including all eligible and available subjects. No a priori power analysis was conducted to determine the sample size. Therefore, the primary aim of this study was to gather preliminary data to explore potential associations and generate hypotheses for future research. Quantitative variables with a symmetrical distribution were summarized by their mean and standard deviation (SD), while continuous variables demonstrating a skewed distribution were expressed using the median (25th percentile, 75th percentile). Qualitative variables were summarized as frequencies and percentages. Group comparisons were conducted using either the Student t-test or Mann-Whitney test, as appropriate, for quantitative variables, and utilizing Pearson’s Chi-square or Fisher’s test, as appropriate, for qualitative variables.

Univariate and multivariable logistic regression analyses were conducted to evaluate the association between main individual variables and high-risk thyroid cytology. Multicollinearity was assessed using Variance Inflation Factors (VIF), with values greater than 5 indicating potential concern. Model fit was evaluated using the Hosmer–Lemeshow omnibus goodness-of-fit test. Variables with a p-value ≤ 0.10 in the univariate analysis were included in the multivariable model. Adjusted odds ratios (OR) with 95% confidence intervals were estimated for each variable. Bivariate correlations between quantitative variables were analysed using Pearson’s correlation coefficients.

The significance level was set at α = 0.05. All statistical analyses were performed using the R statistical software.

## Results

Two hundred-one consecutive patients entered the study, among them, eight were excluded due to inconclusive cytology. Finally, the study included 193 patients, 140 females and 53 males (age 50.3 ± 13.6 years), of whom 16.1% (31/192) were affected by obesity (BMI ≥30 kg/m^2^) (Table 1).

**Table 1:** Main demographic characteristics of the overall population and comparison between group A (benign-low risk thyroid cytology) and group B (high risk-malignant thyroid cytology). Mean$\:\pm\:$SD, median (25th, 75th percentile), BMI = Body mass Index; WC = Waist Circumference; TSH = Thyroid stimulating Hormone; DEHP = bis(2-ethylhexyl) phthalate; BPAF = Bisphenol AF; BPA = Bisphenol A ^#^these data were not available for all patients. Limit of quantification (< LOQ): BPA = 14.47 ng/mL; BPAF = 3.41 ng/mL; DEHP = 6.29 ng/mL. *statistically significant

	All (*n* = 193)	Group A (*n* = 166)	Group B (*n* = 27)	*p*-value
Age (y)	50.3 ± 13.6	50.9 ± 13.3	46.3 ± 15.2	0.110
Sex F/M (%)	140/53 (72.5/27.5)	125/41 (75.3/24.7)	15/12 (55.6/44.4)	**0.033** ^*^
BMI (kg/m^2^)	25.0 (22.0–28.0)	24.6 (22.0-27.7)	27.7 (25.0-30.6)	**0.013** ^*^
BMI ≥ 30 kg/m^2^ yes/no (%) ^#^	31/161 (16.1/83.9)	21/144 (12.7/87.3)	10/17 (37.0/63.0)	**0.001** ^*^
WC (cm) ^#^	88.3 ± 13.9	87.1 ± 13.2	94.8 ± 16.5	**0.008** ^*^
WC (%) ^#^	107/70 (60.5/39.5)	88/62 (58.7/41.3)	19/8 (70.4/29.6)	0.252
TSH (𝜇UI/mL)	1.05 (0.9–2.4)	1.4 (0.8–2.3)	1.8 (0.9–2.5)	0.351
Anti-thyroglobulin antibodies yes/no (%) ^#^	37/90 (29.1/70.9)	27/82 (24.8/75.2)	10/8 (55.6/44.4)	**0.008** ^*^
Anti-thyroperoxidase antibodies yes/no (%) ^#^	34/96 (26.2/73.8)	26/84 (23.6/76.4)	8/12 (40.0/60.0)	0.126
Levothyroxine treatment yes/no (%) ^#^	35/155 (18.4/81.6)	30/133 (18.4/81.6)	5/22 (18.5/81.5)	0.989
DEHP (ng/mL)	8.3 (2.8–32.2)	8.7 (3.0-36.6)	3.7 (2.1–10.0)	**0.020** ^*^
DEHP yes/no (%)	168/25 (87.0/13.0)	147/19 (88.6/11.4)	21/6 (77.8/22.2)	0.122
BPAF (ng/mL)	0.0 (0.0-1.3)	0.0 (0.0-2.5)	0.0 (0.0–0.0)	**0.048** ^*^
BPAF yes/no (%)	58/135 (30.1/69.9)	54/112 (32.5/67.5)	4/23 (14.8/85.2)	0.063
BPA (ng/mL)	0.0 (0.0-0.1)	0.0 (0.0-0.1)	0.0 (0.0–0.0)	0.248
BPA yes/no (%)	51/142 (26.4/73.6)	46/120 (27.7/72.3)	5/22 (18.5/81.5)	0.315
Smoker status present-former/no (%)	47/146 (24.4/75.6)	42/124 (25.3/74.7)	5/22 (18.5/81.5)	0.446
Alcohol consumption yes/no (%) ^#^	57/134 (29.8/70.2)	50/114 (30.5/69.5)	7/20 (25.9/74.1)	0.631
Industrial foodstuffs more or less 3 times/week (%)	24/169 (12.4/87.6)	22/144 (13.3/86.7)	2/25 (7.4/92.6)	0.393
Fruit and vegetables more or less 2 times/day (%)	93/100 (48.2/51.8)	78/88 (47/53)	15/12 (55.6/44.4)	0.409
Physical activity yes/no (%)	91/102 (47.2/52.8)	79/87 (47.6/52.4)	12/15 (44.4/55.6)	0.761
Industrial areas or waste dumping sites yes/no (%) ^#^	58/135 (30/70)	52/144 (31.3/86.7)	6/21 (22.2/77.8)	0.377

Group A included 166 patients and group B included 27 patients, without a significant difference in age between the groups (50.9 ± 13.3 group A, 46.3 ± 15.2 group B; *p* = 0.110).

The percentage of male patients was significantly higher in group B than in group A (44.4% vs. 24.7%, *p* = 0.033).

The prevalence of obesity was significantly higher in group B than in group A (37.0% vs. 12.7%, *p* = 0.001), as well as BMI [27.7 (25.0-30.6) vs. 24.6 (22.0-27.7) kg/m^2^; *p* = 0.013)]. Visceral obesity, defined as WC above the reference range for sex (women < 80 cm, men < 94 cm), was higher in group B than in group A, without reaching statistical significance (70.4% vs. 58.7%, *p* = 0.252), whereas WC was significantly higher in group B than in group A (94.8 ± 16.5 cm vs. 87.1 ± 13.2 cm, *p* = 0.008) (Table 1).

EDCs detected with the highest frequency in the whole population were BPAF [Limit of Quantification (< LOQ): 3.41 ng/mL], BPA (< LOQ: 14.47 ng/mL) and DEHP (< LOQ: 6.29 ng/mL). BPAF was detected in 30.1%, BPA was detected in 26.4% and DEHP in 87.0% of patients, with a median serum concentration of 0.0 ng/mL (range 0.0-1.3), 0.0 ng/mL (range 0.0-0.1) and 8.3 ng/mL (range 2.8–32.2), respectively. DEHP serum levels were significantly higher in group A (8.7 ng/mL, range 3.0-36.6 group A, 3.7 ng/mL range 2.1–10.0 group B, *p* = 0.020), as well as BPAF serum levels (0.0 ng/mL, range 0.0-2.5 group A, 0.0, range 0.0–0.0 group B, *p* = 0.048), conversely BPA levels were comparable between the two groups (0.0 ng/mL, range 0.0-0.1 group A, 0.0 ng/mL, range 0.0–0.0 group B, *p* = 0.248) (Table 1).

EDCs exposure evaluated through self-reported questionnaires did not reveal any difference between group A and B (waste dumping sites 31.3% group A vs. 22.2% group B, *p* = 0.377; industrial foodstuffs 13.3% group A vs. 7.4% group B, *p* = 0.393; fruit and vegetables use 47.0% group A vs. 55.6% group B, *p* = 0.409).

Neither other lifestyle variables (alcohol consumption, physical activity, smoking status) nor thyroid hormones assessment showed significant differences between two groups (alcohol consumption 30.5% group A vs. 25.9% group B, *p* = 0.631; physical activity 47.6% group A vs. 44.4% group B, *p* = 0.761; smoker status 25.3% group A vs. 18.5% group B, *p* = 0.446), except for the prevalence of thyroglobulin antibodies positivity, which was higher in group B (55.6% vs. 24.8%, *p* = 0.008) (Table 1).

In the multivariable analysis, a significant association was found between high-risk cytology and both DEHP exposure (OR 0.72, 95% CI 0.51–0.99, *p* = 0.049) and WC (OR 1.04, 95% CI 1.01–1.08, *p* = 0.027). No significant association was observed with age and region (Table 2). The Hosmer–Lemeshow goodness-of-fit test indicated an adequate model fit (χ² = 20.78, *p* = 0.88). Among all variables included in the model, the highest VIF was 1.24, well below the commonly accepted thresholds (5 or 10), indicating no evidence of multicollinearity. Considering patients with BMI ≥30 kg/m^2^, a significant linear correlation was found between WC and DEHP levels (*r* = 0.40, *p* = 0.034).

**Table 2:** Comparison of main characteristics between group 0 (benign thyroid histology) and group 1 (malignant thyroid histology). Mean$\:\pm\:$SD, median (25th, 75th percentile), BMI = Body mass Index; WC = Waist Circumference; TSH = Thyroid stimulating Hormone; DEHP = bis(2-ethylhexyl) phthalate ^#^these data were not available for all patients. Limit of quantification (< LOQ): DEHP = 6.29 ng/mL. *statistically significant

	Group 0 (*n* = 9)	Group 1 (*n* = 15)	*p*-value
Age (y)	45.1 ± 16.2	42.1 ± 12.4	0.616
Sex F/M (%)	5/4 (55.6/44.4)	7/8 (46.7/53.3)	0.673
BMI (kg/m^2^)	25.7 (23.4–30.0)	28.0 (26.1–30.2)	0.297
BMI ≥ 30 kg/m^2^ yes/no (%)	3/6 (33.3/66.7)	5/10 (33.3/66.7)	1.000
WC (cm)^#^	90.0 ± 17.7	96.6 ± 16.0	0.362
WC (%)^#^	4/5 (44.4/55.6)	11/3 (78.6/21.4)	0.094
TSH (𝜇UI/mL)	1.6 ± 0.7	2.0 ± 1.2	0.365
DEHP (ng/mL)	5.9 (3.0-19.2)	3.7 (1.0-10.7)	0.509
DEHP yes/no (%)	8/1 (88.9/11.1)	11/4 (73.3/26.7)	0.364
Anti-thyroglobulin antibodies yes/no (%)^#^	1/5 (16.7/83.3)	8/3 (72.7/27.3)	**0.027** ^*^
Anti-thyroperoxidase antibodies yes/no (%)^#^	1/6 (14.3/85.7)	6/5 (54.5/45.5)	0.088
Levothyroxine treatment yes/no (%)	1/8 (11.1/88.9)	4/11 (26.7/73.3)	0.364
Smoker status present-former/no (%)	3/6 (33.3/67.7)	1/14 (6.7%/93.3%)	0.090
Alcohol consumption yes/no (%)	5/4 (55.6/44.4)	3/12 (20.0/80.0)	0.074
Industrial foodstuffs more or less 3 times/week (%)	0/9 (0.0/100.0)	2/13 (13.3/86.7)	0.253
Fruit and vegetables more or less 2 times/day (%)	5/4 (55.6/44.4)	8/7 (53.3/46.7)	0.916
Physical activity yes/no (%)	3/6 (33.3/67.7)	7/8 (46.7/53.3)	0.521
Industrial areas or waste dumping sites yes/no (%)	2/7 (22.2/77.8)	4/11 (26.7/73.3)	1.000

Overall, 24 of 193 patients (12.4%) underwent thyroid surgery with a final histological diagnosis of DTC in 62.5% of patients (15/24). The remaining 9 patients (37.5%) received a benign histological diagnosis (follicular adenoma/nodular goiter in 8/24, 33.3%; NIFTP in 1/24, 4.2%). Comparing patients with benign vs. malignant histology (group 0 vs. group 1), although visceral obesity was higher in group 1 (78.6% vs. 44.4%, *p* = 0.094), no significant difference in BMI (25.7 kg/m^2^, range 23.4–30.0 group 0, 28.0 kg/m^2^, range 26.1–30.2 group 1, *p* = 0.297), WC (90 ± 17.7 cm group 0, 96.6 ± 16.0 cm group 1, *p* = 0.362), lifestyle variables, thyroid hormones assessment (TSH 1.6 ± 0.7 µUI/ml group 0, 2.0 ± 1.2 µUI/ml group 1, *p* = 0.365), EDCs serum levels (DEHP 5.9 ng/mL, range 3.0-19.2 group 0, 3.7 ng/mL, range 1.0-10.7 group 1, *p* = 0.509) and exposure was observed. Conversely, the prevalence of anti-thyroglobulin antibodies positivity was significantly higher in group 1 (72.7% vs. 16.7%, *p* = 0.027) (Table 3).

**Table 3:** Simple and multiple logistic regression analysis of variables associated with high-risk thyroid cytology. OR = Odds Ratio; 95%CI = Confidence Interval; DEHP = bis(2-ethylhexyl) phthalate; BPAF = Bisphenol AF; WC = Waist Circumference; Region = comparison between two different populations (Lazio and Campania)

	Univariate Analysis		Multivariable Analysis		
	*OR*	*p-values*	*OR*	*95%CI*	*p-value*
DEHP	0.70	0.024	0.72	0.51–0.99	0.049
BPAF	0.83	0.094	0.90	0.69–1.03	0.244
Sex (F/M)	2.44	0.037	1.42	0.53–3.73	0.478
Age (y)	0.98	0.111	/	/	/
WC (cm)	1.04	0.009	1.04	1.01–1.08	0.027
Region	1.12	0.833	/	/	**/**

A molecular analysis was performed on cytology specimens, available in 43 subjects. The following mutations were highlighted: two BRAF mutations, one in a patient with TIR5 cytological diagnosis who refused surgery, and one in a patient with TIR4 cytology, not available histological diagnosis; one HRAS mutation in a patient with TIR3A cytology, who is still on ultrasound follow-up. The remaining 40 cytology specimens showed a wild-type genotype.

## Discussion

The results of the current study show that determinants of obesity (BMI and WC) were significantly higher in patients with cytological risk of thyroid carcinoma compared to those with benign cytology, supporting the hypothesis of a potential role of obesity and, more specifically, of visceral fat in the development of DTC. These findings were confirmed by multivariable analysis that showed a significant association between high-risk cytology and WC. It is noteworthy that BMI is independently associated with a risk factor for DTC development, which could be consistent with the pro-inflammatory action of visceral adiposity, reported in previous studies [32, 37, 38]. WC is considered the best parameter of android obesity and visceral fat, and it is preferred to BMI, which does not discriminate lean from fat mass [39]. Nowadays, visceral fat is recognized as an endocrine organ, secreting cytokine and promoting inflammation status, being involved in cardiovascular risk and tumour development [32, 40, 41]. Potential mediators of this complex process may include higher levels of insulin and leptin, which have been implicated in cell growth and proliferation, as well as angiogenesis [41, 42].

Although exposure to environmental pollutants has been associated with the impairment of thyroid function, data on the role of EDCs in DTC development are still lacking and those present in the literature are not univocal.

A recent study from Marotta and coworkers evaluating the role of lifestyle and EDCs exposure in thyroid cancer development showed a significant association between thyroid carcinoma and BPA exposure, but only in the subgroup of patients with overweight/obesity, suggesting an interaction between BPA exposure and adipose tissue excess in promoting thyroid carcinogenesis [16]. In analogy with the previous study, our study population shows a relationship between DEHP serum levels and WC, albeit BPAF and DEHP serum levels were significantly higher in patients with low-risk/benign thyroid cytology, when considering the subgroup of patients with obesity. These findings suggest a possible interaction between EDCs exposure and adipose tissue excess that might contribute to DTC risk, although further studies are needed. DEHP is a highly lipophilic compound, with a log P value of 7.60 and, even if it can be metabolized to MEHP, it could concentrate more than the other chemicals in adipose tissues and be released from fat depots toward the bloodstream. DEHP is included as substance of very high concern according to Article 57(f) of Regulation (EC) No 1907/2006 of the European Parliament and of the Council. MEHP has been proved to be highly toxic and biologically active on thyroid as demonstrated by several scientific researches on animal studies [43, 44].

However, evidence from human epidemiological studies evaluating the association between EDCs exposure and DTC risk is extremely scarce and inconsistent. A previous cross-sectional study of 178 subjects, including 53 patients with DTC, 60 patients with nodular goiter, and 65 healthy controls from China, showed a significantly positive association between urinary BPA concentrations and the prevalence of DTC [14]. Recently, these data were partially confirmed by Zhang et al. in a case-control study, including 222 patients and comparing urinary levels of bisphenols (BPS, BPF, and BPS) between patients with DTC and healthy controls. Indeed, they found higher urinary levels of BPF in patients with DTC, while BPA and BPS were significantly lower [15], thus suggesting that different types, levels, and time exposure to EDCs should have a role in promoting the oncogenic processes in thyroid nodules. Finally, a study by Carli et al. assessed co-exposure to phthalates and BPA in the Italian population, including people from the north, centre and south of Italy, with a total of 898 women equally distributed demographically. The results showed a positive correlation between DEHP levels and BMI, especially in southern Italy’s population, while the BPA exposure was significantly higher in the north of Italy [45].

Moreover, previous studies in mice models showed that BPA and DEHP exposure seem to induce oncogenic mutations at the cellular level, which may provide biological plausibility in the induction of proliferative mechanisms, probably linked to the development of thyroid carcinoma, but cannot be directly extrapolated to humans [46]. In our cohort, the assessment of the mutational profile did not show any significant difference in the prevalence of gene mutation between the two groups, probably due to the small number of specimens.

Lifestyle, including diet, physical activity, smoking habit, and alcohol consumption, has been widely found to be involved in cancer development and progression and it has also been advocated in the development of DTC, even though some of these variables seem to act in opposite ways than expected and data are not univocal [20, 47, 48].

In our cohort, the evaluation of lifestyle variables did not differ significantly between the two groups with different cytological risks of DTC.

Literature data on alcohol consumption and DTC risk are controversial. Most of the studies suggest a protective role of alcohol consumption in the development of DTC. A meta-analysis of observational studies found that alcohol intake decreased the risk of thyroid carcinoma, unlike most other types of cancer [49]. In the current study, the alcohol consumption was not significantly different between groups.

The association between smoking habit and DTC risk is still a matter of debate, being evidence more suggestive of a protective role. Smokers appear to have a lower risk than non-smokers and former smokers of developing thyroid cancer, probably due to the lower TSH levels and BMI of smokers [50]. In a cohort study of 96,855 Korean adults, current smoking was associated with a reduced risk of incident thyroid carcinoma in men but not women. This association was also observed after adjustments for TSH levels and BMI as potential mediators [51]. In a later huge cohort study of 10 million Koreans, the protective role of smoking against thyroid carcinoma was confirmed and the risk of developing DTC was inversely associated with smoking and alcohol consumption, with a sub-multiplicative interaction between smoking and alcohol intake [52]. A recent Chinese study indicated that former smoking was inversely associated with thyroid carcinoma occurrence in males and the reduction in the occurrence was also confirmed for both former and current smokers with higher smoking intensity, duration, and cumulative dose [53]. A previous study in a cohort of postmenopausal women suggested that current smoking and having higher pack-years of exposure were associated with a modestly reduced risk of thyroid carcinoma, whereas alcohol consumption did not appear to affect risk [54]. In the present study, no difference either in smoking habits or in TSH levels was observed between groups.

The association between physical activity and thyroid carcinoma remains poorly understood. Long-term recreational physical activity, practiced since childhood, was found to reduce DTC risk. However, the underlying mechanisms are not yet entirely elucidated [55]. Similarly, long-term physical activity was found to reduce DTC risk in normal-weight and underweight women [56].

Moreover, a little case-control study conducted in South Italy showed that daily walking duration was associated with a lower risk of thyroid carcinoma [57]. In our cohort, we didn’t find any difference in physical activity when comparing patients of the two groups.

Furthermore, our findings showed that the prevalence of nodular disease is higher in females, confirming the literature data [33]; conversely, a significantly higher prevalence of male sex was found in patients with high-risk/malignant cytology, supporting the postulate that we should look more carefully thyroid nodules in males, given their more aggressive behaviour, poor prognosis, and risk of recurrence [58, 59].

As expected, we found a higher prevalence of anti-thyroglobulin antibodies in patients with high-risk/malignant cytological diagnoses that were confirmed in the subgroup of patients with malignant histology. The association between autoimmune thyroiditis and thyroid carcinoma has been widely described in the literature, with the most frequent association observed with papillary thyroid carcinoma. To date, the hypothesis receiving the greatest consensus is that this co-occurrence may reflect common pathophysiological origins, more than a causal link [60].

Overall, the current study partially confirmed the results of previous studies in larger cohorts of patients and our findings are consistent with, and add exploratory support to, the hypothesis of a possible interaction between EDCs exposure and adipose tissue excess in DTC development although they must be viewed with caution. However, it does have some limitations to be acknowledged: first, the small number of patients in the high-risk cytological group and as a consequence the small rate of thyroid surgery and histological diagnoses. This reflects the performance of thyroid nodule work-up in the involved centers; second, the lack of a control healthy group, but the low-risk cytological group represents an internal control to compare with the high-risk group. Given the exploratory and cross-sectional nature of the study, the observed associations cannot be interpreted as causal. The proposed mechanisms are speculative and intended only to provide a possible biological rationale to be tested in future studies.

## Data Availability

No datasets were generated or analysed during the current study.
